# Utilization of Cellulose to Its Full Potential: A Review on Cellulose Dissolution, Regeneration, and Applications

**DOI:** 10.3390/polym13244344

**Published:** 2021-12-12

**Authors:** Sanjit Acharya, Sumedha Liyanage, Prakash Parajuli, Shaida Sultana Rumi, Julia L. Shamshina, Noureddine Abidi

**Affiliations:** Department of Plant and Soil Science, Fiber and Biopolymer Research Institute, Texas Tech University, Lubbock, TX 79409, USA; sanjit.acharya@ttu.edu (S.A.); sumedha.liyanage@ttu.edu (S.L.); prakash.parajuli@ttu.edu (P.P.); shaida-sultana.rumi@ttu.edu (S.S.R.); jshamshi@ttu.edu (J.L.S.)

**Keywords:** cellulose, dissolution, non-derivatizing solvents, coagulation, applications, materials

## Abstract

As the most abundant natural polymer, cellulose is a prime candidate for the preparation of both sustainable and economically viable polymeric products hitherto predominantly produced from oil-based synthetic polymers. However, the utilization of cellulose to its full potential is constrained by its recalcitrance to chemical processing. Both fundamental and applied aspects of cellulose dissolution remain active areas of research and include mechanistic studies on solvent–cellulose interactions, the development of novel solvents and/or solvent systems, the optimization of dissolution conditions, and the preparation of various cellulose-based materials. In this review, we build on existing knowledge on cellulose dissolution, including the structural characteristics of the polymer that are important for dissolution (molecular weight, crystallinity, and effect of hydrophobic interactions), and evaluate widely used non-derivatizing solvents (sodium hydroxide (NaOH)-based systems, *N*,*N*-dimethylacetamide (DMAc)/lithium chloride (LiCl), *N*-methylmorpholine-*N*-oxide (NMMO), and ionic liquids). We also cover the subsequent regeneration of cellulose solutions from these solvents into various architectures (fibers, films, membranes, beads, aerogels, and hydrogels) and review uses of these materials in specific applications, such as biomedical, sorption, and energy uses.

## 1. Introduction

### 1.1. The Sustainability Aspects of Cellulosic Materials

Cellulose is the most abundant renewable natural polymer on Earth, so it is of substantial economic importance [1]. It is the main constituent of plant fiber, which makes one third of all plant materials on average, although the amount of cellulose greatly varies from source to source. For instance, cotton fiber is the purest form of plant cellulose, with a cellulose content as high as 90%, while the cellulose content in woody biomass ranges from 40% to 50% [2,3]. Historically, cellulose sources have primarily been used as fuel, reinforcements for building construction (e.g., wood), and clothing (e.g., cotton) [4]. Recent progress in the chemistry of cellulose allowed for new emerging opportunities for this biopolymer in, for instance, the production of biofuels [5] and the preparation of biopolymeric products (films, aerogels, sponges, etc.), hitherto predominantly produced from oil-based synthetic polymers [6,7,8].

Synthetic polymers are made from petroleum, which is not practically renewable and its reserve is dwindling. Since synthetic polymers are largely non-biodegradable, there are growing concerns about the accumulation of plastics in landfills and natural habitats, and the potential release of additives present in plastics that pose hazards to humans and animals [9,10,11]. In addition, there are concerns about the high emission of greenhouse gases from petroleum-based industries, and materials prepared from bio-based renewable resources are considered to be greener alternatives [12].

By virtue of being the most abundant, renewable, biodegradable, and biocompatible polymer, cellulose is obviously a promising resource for the preparation of green products for different applications. In this regard, the benefits of the use of cellulose over other biopolymers are numerous. Firstly, cellulose is considered to be a virtually inexhaustible and relatively cheap resource [1]. Secondly, because the major sources of cellulose are trees, which are generally grown in comparatively marginal lands, cellulosic feedstocks do not compete for land and water resources that could otherwise be devoted to food/feed production (e.g., starch and soy protein [13]). There is also a vast opportunity for the utilization of agricultural wastes as cellulosic sources [14]. Among other plant-based carbohydrates, cellulose exhibits the highest degree of resistance to degradation [15]. This property is advantageous for the required durability in many consumer products while still allowing for biodegradability once disposed.

### 1.2. Limitations in Uses of Cellulose

Despite the numerous advantages of cellulose as a replacement for traditionally made petroleum-based plastics, the use of cellulose to its full potential in its chemically unmodified form is limited. In contrast to commonly used thermoplastics, cellulose cannot be melted without structural disintegration and is insoluble in most common solvents. Over the last hundred years of continuous research on cellulose dissolution [16], various solvents and/or solvent systems have been discovered and utilized for this purpose [4,17]. These include alkaline systems (e.g., sodium hydroxide-based systems), non-aqueous polar aprotic systems (e.g., *N*,*N*-dimethyl acetamide/lithium chloride), mineral acids (e.g., phosphoric acid), ionic liquids (ILs), and deep eutectic solvent solvents (DESs). However, cellulose dissolution still remains an active area of research because it is the only viable option for processing the biopolymer in its natural form without derivatization. There are many fundamental aspects such as solvent–cellulose interactions and implementation challenges such as scaling up the dissolution process and solvent recovery that remain to be fully resolved before cellulose-based products conquer the marketspace. In this review, we build on the existing knowledge on cellulose dissolution (excluding biological pretreatments that are viewed as too long to be economically viable), discuss different types of produced cellulose materials, and examine the applications of these materials in different areas.

## 2. Structural Characteristics of Cellulose Important for Dissolution

**Figure 1:**
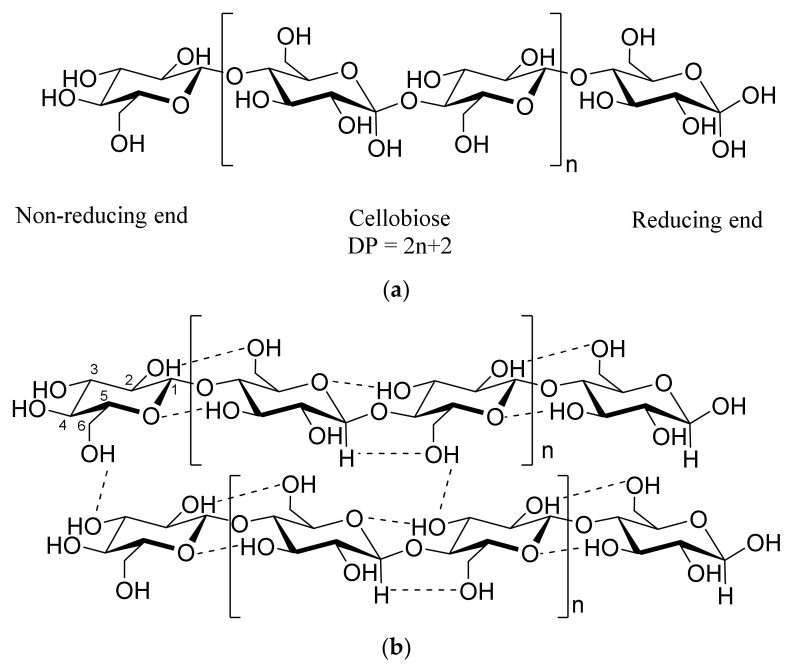
Chemical structure and hydrogen bonding in cellulose: (**a**) basic chemical structure of a cellulose chain; (**b**) intra- and interchain hydrogen bonding in cellulose.

Cellulose is biosynthesized by living organisms (plants, some species of bacteria, algae, and tunicates—the only known animals capable of biosynthesizing cellulose) as a linear homopolymer of *β*-D glucose monomers [18]. The polycondensation reaction between the hydroxyl groups at C1 of a glucose unit and C4 of the neighboring glucose unit leads to the covalent linking of monomers by 1 → 4 glycosidic bonds (so-called “beta linkages”) [18]. Although recently debated [19], cellobiose, a dimer of glucose, is considered to be a repeating unit of cellulose polymer because two neighboring glucose units are rotated 180° with respect to each other along the fiber axis in a polymer chain (Figure 1a) [20]. Typically, cellulose is a high-molecular-weight polymer. The chain length is expressed by the number of constituent glucose monomers in a polymer chain (i.e., degree of polymerization (DP)). The DP of cellulose varies from source to source and depends on the cellulose extraction processes. Generally, it is 10,000 and 15,000 for wood and cotton cellulose, respectively [6,21].

The abundance of hydroxyl groups in cellulose (each monomeric glucose contains three hydroxyl groups) leads to the establishment of extensive hydrogen bonding within and between the polymeric chains. Intra-chain hydrogen bonding, formed between O(3)–H…O (ring oxygen) and O(2)–H…O(6) (Figure 1b), stabilizes the glycosidic bonds and makes the structure rigid. Inter-chain hydrogen bonding occurs between O(3)–H…O(6) and nonconventional CH…O-type hydrogen bonding (Figure 1b). Stacking forces (mainly Van der Walls forces) are largely responsible for the parallel stacking of multiple cellulose chains causing the formation of sheets [22,23,24]. This leads to the hierarchical organization of cellulose giving rise to supramolecular structure, with cellulose chains forming an elementary fibril, elementary fibrils forming a microfibril, and ultimately microfibrils making a native cellulose fiber found in the nature (e.g., wood fiber and cotton fiber) [25]. An elementary fibril comprises 36 cellulose chains and has a diamond-shaped cross-section with dimensions of 3.5 × 5.3 nm [26,27]. These elementary fibrils further assemble into a larger dimensional microfibril of 5–50 nm in diameter and several micrometers in length. Ultimately, several microfibrils are bundled together to form a native cellulose fiber [18].

**Figure 2:**
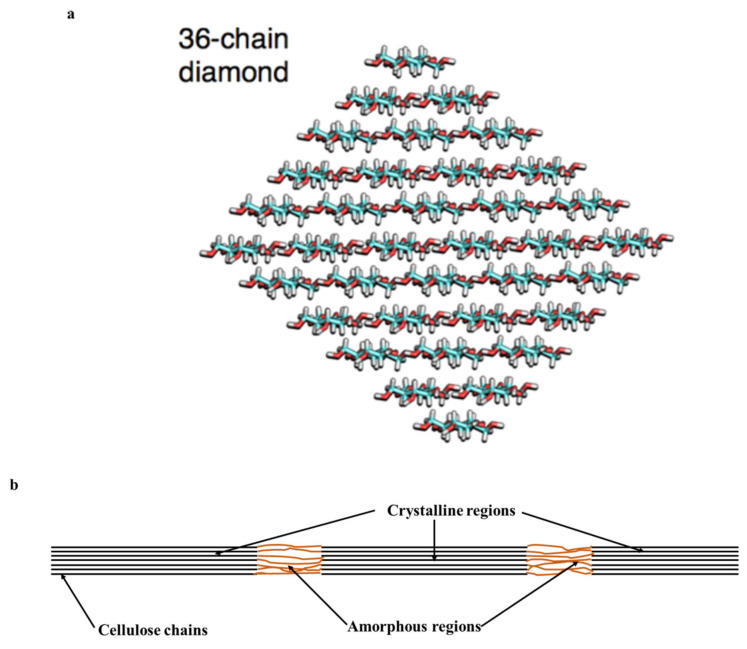
Organization of cellulose: (**a**) prevailing 36-chain model of cellulose elementary fibril. Adapted with permission from [30], © 2021 American Chemical Society; (**b**) schematic representation of cellulose microfibril showing crystalline and amorphous structure.

However, some studies have suggested alternate models of the arrangement of cellulose chains in the microfibrils. For example, one study hypothesized that spruce wood microfibrils might comprise 24 cellulose chains [28]. A recent study analyzed X-ray diffraction (XRD) patterns and nuclear magnetic resonance (NMR) spectra and compared them with computational data [29]. It was found that an 18-chain model provided a better fit than a 24-chain model even though a good agreement between experimental and computed diffractograms and spectra was achieved with both models [29]. This work alluded to the fact that cellulose microfibrils in higher plants may be smaller than the commonly consented 36-chain model (Figure 2a).

**Figure 3:**
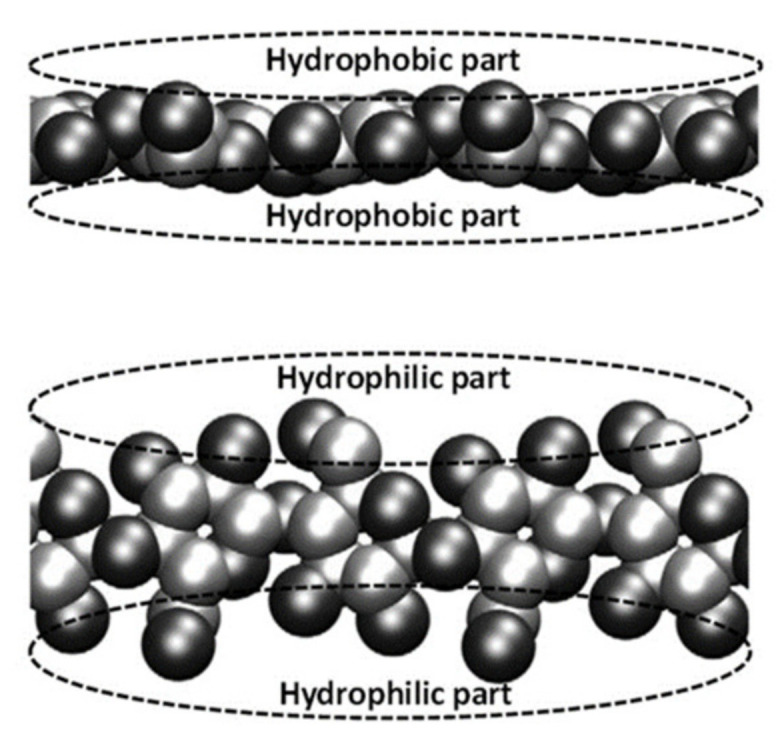
A Van der Waals surface representation of cellulose chain showing hydrophobic and hydrophilic parts. Oxygen atoms are colored black and non-polar carbon atoms are shaded grey in this representation. Reprinted from [33] © 2021 with permission from Elsevier.

Even though the specific packing of cellulose is yet to be resolved, it is well-established that the extensive intra- and interchain hydrogen bonding and Van der Walls forces contribute to highly ordered (crystalline) regions, albeit with alternate regions of lesser order (amorphous regions) (Figure 2b) [27,30]. Such a complex, extensively hydrogen-bonded, highly crystalline supramolecular structure of cellulose offers little accessibility to solvents and is widely considered to be responsible for its insolubility in water and common solvents [31,32]. Furthermore, this is also one of the reasons for cellulose resistivity towards microbial degradation because enzymes only have access to fraction of cellulose chains on the microfibril surfaces [30]. Although it is rarely emphasized while discussing the structure of cellulose, cellulose fibrils are amphiphilic in nature; they contain both hydrophobic and hydrophilic characters [27]. The Van der Waals surface representation of the cellulose chain shown in Figure 3 helps visualize amphiphilic character of cellulose. Because all three hydroxyl groups of a glucose monomer ring are equatorial and the hydrogen atoms of C–H bonds are axial, cellulose has amphiphilic property with hydrophilic character in the equatorial direction and hydrophobic character in the axial direction [33].

For the dissolution of cellulose to take place, it is necessary to disassemble its supramolecular structure and separate the cellulose chains, ideally without or with the nominal breakage of glycosidic bonds. It is agreed among experts in the field that, theoretically, cellulose dissolution happens through the breakage of native hydrogen-bonded network, especially in the crystalline regions [32]. The hydrogen-bonded network is impacted by traditional derivatizing solvents (e.g., in viscose process) when cellulose is chemically modified through reactions with hydroxyl groups, which would derivatize them and therefore disrupt the hydrogen bonding [34]. On the other hand, non-derivatizing solvent or solvent systems are capable of disrupting the native hydrogen-bonding network of cellulose by establishing new hydrogen bonds with hydroxyl groups of cellulose [35,36,37], thus destroying its crystalline structure [38]. Since crystalline cellulose can be easily visualized using polarizing light microscopy, the technique is routinely used to examine cellulose dissolution [38,39,40].

Some non-derivatizing solvent systems, specifically molten inorganic salt hydrates (e.g., ZnCl_2_·3H_2_O), destroy the native hydrogen bonding of cellulose via the coordination of metal ions present in the medium and the hydroxyl groups of the cellulose [36]. In addition to the need to break the hydrogen-bonding of cellulose, some researchers have stressed that hydrophobic interactions also need important considerations in respect to cellulose dissolution [4].

## 3. Important Factors in Cellulose Dissolution

### 3.1. Molecular Weight

The molecular weight of a polymer is a key parameter and is inversely related to the entropic driving force contribution for dissolution. Therefore, the inherent high molecular weight of cellulose results in a decreased solubility in the solvents due to decreased entropic gain, especially translational and configurational in the dissolution process [41]. The importance of the molecular weight of cellulose in its dissolution is evident—cellulose is insoluble in water but glucose (the basic unit of cellulose), cellobiose (the repeating unit of cellulose), and oligocelluloses with degrees of polymerization of less than 10 are water-soluble [42,43]. In order to overcome entropic penalties resulting from the high molecular weight of cellulose, the dissolution system should involve favorable solvent–polymer interactions [44].

Decreasing the molecular weight of cellulose is an efficient thermodynamic action that helps in cellulose dissolution in thermodynamically “not so powerful solvents” such as aqueous NaOH systems [45]. Several studies have shown that decreasing the molecular weight of cellulose could be an effective strategy to achieve its enhanced dissolution [46,47,48]. Trygg and Fadim employed acid hydrolysis and showed that hydrolyzed samples with significantly lower molecular weights were more readily dissolved in the NaOH/urea/water system [47]. Even though a significant decrease in molecular weight of cellulose may generally lead to a decrease in the mechanical strength of regenerated cellulose materials [49], seeking the improved dissolution of cellulose via decreasing its molecular weight could still be an effective strategy in specific situations such as those where high mechanical strength is not a requirement for the final cellulosic products. This strategy could also be beneficial when cellulose dissolution is employed as a pretreatment to enhance enzymatic catalysis for biofuel production [5,50,51].

### 3.2. Crystallinity

Cellulose is a highly crystalline polymer that offers little accessibility of solvent to the inside of the polymeric network [1,34]. Generally, cellulose crystallinity is considered to be an important factor in its dissolution and affects cellulose dissolution level for high molecular weight cellulose [32,52]. Good access of the solvent is necessary for the eventual dissolution of cellulose to occur. Cellulose dissolution involves decrystallization followed by the disentanglements of polymer chains [53].

Experimental evidence from numerous studies on the effect of cellulose crystallinity has been contradictory [52,54]. Several studies have shown that pretreatments that reduce the crystallinity and crystallite size of cellulose substrates result in the enhanced dissolution of cellulose in *N*,*N*-dimethyl acetamide/lithium chloride (DMAc/LiCl) solvent systems [55,56]. Similarly, in another solvent system (NaOH/urea/water), cotton linter pulp with higher molecular weight but lower crystallinity was more readily dissolved than the pulp, having half the molecular weight but higher crystallinity, under identical dissolution conditions [57]. On the contrary, Ishi and coworkers [58] reported that under identical dissolution conditions, ball-milling pretreatment that almost fully destroyed cellulose crystallinity did not improve its dissolution in DMAc/LiCl while solvent-exchange pretreatment that did not alter the crystalline structure of the polymer facilitated its dissolution. Parviainen et al. also demonstrated that two different pulps with similar crystallinity dissolved differently under the same treatment, although other factors such as molecular weight could play a role [52]. More recently, in a study using a phenomenological method, Ghasemi and coworkers demonstrated that the effect of cellulose crystallinity may or may not be a factor. If decrystallization is the rate-determining step, the decreased crystallinity of cellulose fiber helps in dissolution; when the chain disentanglement is the rate-determining step, fiber diameter is more important than crystallinity [59].

### 3.3. Hydrophobic Interactions

A group of researchers highlighted the “clear amphiphilic” properties of cellulose (Figure 3) and hydrophobic interactions as important factors in governing cellulose solubility [4,33]. Indeed, a free energy simulation study that used cellulose oligomers as model compounds estimated the overall contributions of hydrophobic stacking and hydrogen bonding to the insolubility of crystalline cellulose as 2 kcal/(mol·residue). It was also determined that hydrophobic association favored a crystal-like structure over a hypothetical solution state, and that contributions of hydrophobic interactions were about eight times stronger than those of hydrogen bonding [60]. The authors emphasized that hydrophobic interactions should not be overlooked in the dissolution process. Similarly, Medronho and others [4,33,61] rationalized the improved dissolution of cellulose in ILs and NaOH/urea/water by reducing cellulose hydrophobic interactions in this solvent system. Thus, in ILs, the hydrophobic interactions between cellulose chains are altered by amphiphilic cations, whereas the addition of urea to NaOH/H_2_O decreases solvent system polarity and weakens the hydrophobic interactions in cellulose, leading to improved dissolution [4,33,61].

## 4. Cellulose Solvents

Cellulose solvents are broadly classified into derivatizing and non-derivatizing solvents. In derivatizing systems, cellulose dissolution is achieved via the initial formation of ester, ether, or acetal [33,62] After cellulose derivatives are dissolved and shaped (i.e., in form of filaments or films), the cellulose derivative is subsequently converted back to cellulose. For example, industrially important rayon and Cellophane are produced by exploiting derivatizing system known as “viscose” (NaOH + CS_2_) [62,63]. In a typical viscose process, cellulose xanthate derivative, which is soluble in aqueous NaOH, is formed. After the shaping and regeneration of a cellulose xanthate solution in NaOH, the cellulose xanthate is converted back into cellulose [1].

The second type of solvent systems, “non-derivatizing” ones, are capable of dissolving cellulose without chemical modification. The dissolution of cellulose in non-derivatizing solvents occurs via the disruption of the cellulose hydrogen-bonding network [33]. A number of direct cellulose solvents have been reported [34,37,64] and include alkaline systems (NaOH–water), *N*,*N*-dimethyl acetamide/lithium chloride (DMAc/LiCl), *N*-methylmorpholine-*N*-oxide (NMMO), and numerous ILs [16]. Every so often it is claimed that aqueous solutions of mineral acids are able to dissolve cellulose. However, though acids swell cellulose, dissolution can only be achieved using highly concentrated mineral acids. The dissolution is associated with severe, if not complete, cellulosic chain degradation (breaking 1,4-glycosidic bonds) happening over time. Thus, the treatment of raw cotton cellulose with 5 N HCl for 15 min, at a relatively low temperature of 5 °C, resulted in a drop of DP from 3200 to 2100 [65]. The same treatment conducted for 30 min produced cellulose with a DP of almost twice lower than that of starting material, 1780 [65]. In the follow-up sections, we review the non-derivatizing solvent systems for cellulose in more detail.

### 4.1. Aqueous Sodium Hydroxide (H_2_O-NaOH)-Based Solvent Systems

The discovery of cellulose treatment with a solution of sodium hydroxide (mercerization) and the viscose process were the starting points in the use of NaOH in the cellulose industry [45]. Since then, extensive research has been conducted to explore cellulose dissolution in aqueous NaOH, either alone or with additives (e.g., urea) [37,66,67,68]. Enormous research interest in NaOH-based systems as promising solvents for cellulose stems from their attributes such as low cost, availability, and ease of recovery [45].

Initially, it was reported that the dissolution of cellulose in NaOH occurs at a narrow range of NaOH concentration (7–10%) and a relatively low temperature (<10 °C) [69]. Later, studies suggested that the dissolution capacity of the aqueous NaOH is increased at sub-zero temperatures (0 °C) [66,70]. The dissolution conditions depend on different factors such as molecular weight and crystallinity [71]. Thus, cellulose solubility is limited to celluloses of relatively low DP (<250) and crystallinity, and cellulose solubility is limited to 5% [72]. However, intensive research studies to improve cellulose dissolution have been conducted using different slightly modified solvent systems, leading to more efficient dissolution than the binary NaOH–water system. Despite this, the dissolution ability is not up to par compared to other more recently developed solvents that are often preferred to aqueous NaOH for cellulose dissolution [45]. The major drawback of aqueous NaOH as a solvent for cellulose is that it only dissolves cellulose in a limited range of NaOH concentrations at low temperatures [73]. Moreover, the cellulose solubility in NaOH is relatively low, the resulting solution is of low stability, and the prepared materials (e.g., fibers) exhibit moderate mechanical properties [45].

The exact mechanism of the cellulose–NaOH interaction has not yet been established, and views of the mechanism are rather contradictory [45]. A somewhat common understanding is summarized as follows. The NaOH molecules in the aqueous system form NaOH hydrates at low temperatures. These NaOH hydrates disrupt closely packed cellulose by establishing hydrogen bonding with one or two hydroxyl groups of each anhydroglucose unit of the cellulose molecules [4,37]. Many pieces of literature have reported that Na^+^ breaks the O2–H…O6 hydrogen bond [74,75]. However, Xiong et al. reported that OH^−^ (being a strong hydrogen-bond acceptor) breaks the intra- and intermolecular hydrogen bonds of cellulose by forming a hydrogen bond with water and cellulose hydroxyl groups, whereas the role of Na^+^ cation is limited to stabilizing the solution by preventing the cellulose chains from coming closer to each other (although it can also form a complex with cellulose) [76]. The narrow working concentration range of NaOH could be explained by the concentration-dependent size of NaOH–water hydrates. Supposedly, the too-large hydrodynamic radii of the NaOH hydrates at lower concentrations of NaOH prohibit the NaOH hydrates from penetrating and diffusing inside the densely packed crystalline fibrillar network of cellulose. The improved dissolution of cellulose in the NaOH–water system when additives such as urea are added is attributed to the formation of an inclusion complex via the possible self-assembly of urea hydrates at the surface of the NaOH–cellulose complex. The roles of urea and thiourea as hydrogen-bond donors and acceptors to prevent the reassociation of cellulose chains to increase the stability of the solution have been emphasized in numerous studies [37,77,78,79,80].

#### Strategies for Improvement of Cellulose Dissolution in NaOH-Based Solvents

Despite the apparent simplicity, low cost, and recyclability of the aqueous NaOH–solvent system, its utilization as a solvent for cellulose is limited. This is because cellulose dissolution in the NaOH–water system is only partial and is limited to a specific concentration range of NaOH. Different techniques have been applied to improve cellulose dissolution in aqueous NaOH solvents. Additives such as urea, thiourea, zinc oxide (ZnO), and polyethylene glycols (PEGs) of various molecular weight have been shown to improve both the cellulose solubility and stability of the resulting solution. Different pretreatments of cellulose such as mechanical (using, e.g., hydrothermal treatment [81] and steam explosion [82,83]), chemical (using, e.g., ethanol and hydrochloric acid) [47], and enzymatic (using, e.g., cellulase enzyme) [84] are used. These pretreatment techniques have been shown to be effective in the improvement of cellulose dissolution in aqueous NaOH-based solvent systems and to promote the accessibility of solvents, thereby creating favorable polymer–solvent interactions [45]. A few examples of pretreatments for cellulose dissolution in aqueous NaOH-based solvent systems are summarized in Table 1.

### 4.2. N,N-Dimethylacetamide (DMAc)/Lithium Chloride (LiCl) System

McCormick et al. first reported the direct dissolution of cellulose in the DMAc/LiCl solvent system in 1979 [94]. Today, the DMAc/LiCl solvent system plays a crucial role in cellulose dissolution [95] and is considered to be a powerful solvent in cellulose chemistry [96,97,98]. The DMAc solvent can be compared with other solvents in terms of its cost and recyclability [99], although DMAC is classified as harmful by inhalation and skin contact according to the criteria of Directive 67/548 EEC and thus not environmentally-friendly [100].

DMAc is used in combination with LiCl. Different studies have used different amounts of LiCl in DMAc, from 5% to 13% [101,102,103,104], and 8% (w:v) of LiCl in DMAc is the most often used system [7,8,105]. The DMAc/LiCl solvent system is used not only for the preparation of cellulose materials but also for cellulose derivatizations. Furthermore, it is widely used for analytical purposes, specifically for cellulose molecular weight estimation by gel permeation chromatography (GPC) [106,107,108,109,110,111,112,113]. A wide range of cellulose dissolution conditions in terms of the temperature, the time of dissolution, and the concentration of cellulose have been employed (Table 2). One of the major drawbacks of the DMAc/LiCl solvent system is its highly hygroscopic nature because both components DMAc and LiCl are highly hygroscopic and the dissolution ability of the solvent is seriously diminished with the presence of water [102]. Another important drawback that needs to be considered is cellulose degradation due to the formation of keteniminium cation, a highly reactive intermediate formed in the binary DMAc/LiCl at elevated temperature (>85 °C) that induces the cleavage of glycosidic bonds [104].

The stability of cellulose solution in DMAc depends on several factors, such as the amount of LiCl, cellulose content, the time of solution storage, and the presence of water [101,102] (which reduces the solution quality and results in polymer aggregation). The amount of water and LiCl in a solvent system can be described as a critical parameter influencing the dissolution [101,102]. In a study by Chrapava et al., it was demonstrated that the number of water molecules should not exceed two for each molecule of LiCl to achieve the complete dissolution of cellulose [101]; other studies have claimed the need to dehydrate the DMAc/LiCl system to 0.9 wt% or less of water [102,104]. Mechanistically speaking, water is absorbed into the solvation shell of Li^+^ [104], resulting in the hydrolysis of DMAc and triggering additional water absorption into already wet DMAc. Water accumulated in the solvent system limits the availability of the solvation shell of Li^+^ to cellulose complexation [104]. Different procedures have been suggested to dry LiCl, such as flame-drying. DMAc is dried by distillation followed by the addition of a water-trapping agent in order to keep the solvent system water-free [102].

**Figure 4:**
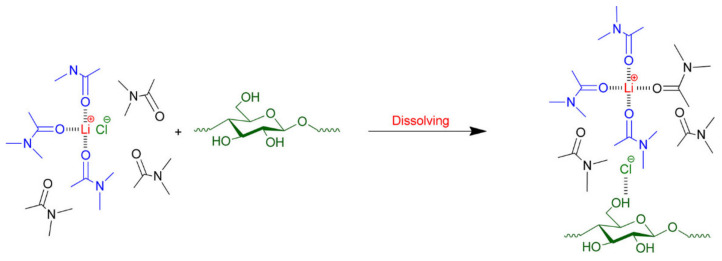
Interaction of cellulose with Li^+^ cations, Cl^−^ anions, and DMAc during its dissolution in the DMAc/LiCl solvent system, proposed by Zhang et al., 2014. Reprinted with permission from [35] © 2021 American Chemical Society.

There have been numerous studies regarding the mechanism of cellulose dissolution in DMAc/LiCl [114]. It is postulated that the intense interaction between chloride anions and cellulose disrupts the cellulose hydrogen-bonding network. Indeed, it has been reported that about 80% of the dipole–dipole interactions between DMAc and cellulose come from chloride anion–cellulose interactions. Moreover, the Li^+^ cations are further solvated by free DMAc molecules, and about 10% of the dipole–dipole interactions between DMAc and cellulose are contributed by cellulose/macro-cation complexes ([Li^−^(DMAc)_x_]) [86,88]. Zhang et al. recently revisited the interactions of cellulose with DMAc and LiCl [35]. In this solvent system, Li^+^–Cl^−^ ion pairs are separated when cellulose is dissolved. The intermolecular hydrogen bonding of cellulose disrupts with the splitting of Li^+^–Cl^−^ ions because a strong hydrogen bond is formed between the hydroxyl protons of cellulose and the Cl^−^ ions of the salt. There is no interaction between the carbonyl group in DMAc molecules and the hydroxyl proton of cellulose. This mechanism is depicted in Figure 4.

#### Strategies for Improvement of Cellulose Dissolution in DMAc/LiCl Solvent System

There are different approaches for achieving the better dissolution of cellulose in DMAc/LiCl, including cellulose pretreatment or cellulose activation. Activation causes structural and morphological changes in cellulose, creating a favorable environment for dissolution and facilitating DMAc solvent diffusion into cellulose macromolecules [115].

The most common activation strategy is a solvent-exchange method in which cellulose is swelled up with a polar medium, usually water [97,101,116]. After removing the excess of water, cellulose is rinsed with acetone [101,116] or methanol [97] and then dried, followed by dissolution in DMAc/LiCl at room temperature [101,102,116]. Water swelling enhances the accessibility of DMAc into the cellulose macromolecular structure [117], eventually resulting in better dissolution. Roder et al. and Hu et al. reported cellulose activation in liquid ammonia and low-concentration acetic acid [116,118], and Raus et al. introduced a dioxane-based activation method suitable for large cellulose quantities [119].

Another way of activation is swelling in water followed by freeze-drying, which does not cause a significant change in its crystalline structure but instead is associated with increasing cellulose porosity, void fraction, and surface area [8]. This method has been found to be suitable for both low-molecular-weight and high-molecular-weight cellulose. During freeze-drying, water is removed from a solid to a vapor phase, and the void spaces occupied by frozen water are retained in the freeze-dried cellulose. The created environment allows for the faster diffusion of the solvent into the cellulose’s internal molecular network, thus leading to enhanced dissolution. Acharya et al. demonstrated the effect of freeze-drying pretreatment on cotton cellulose dissolution. The same phenomenon was also reported by Hu et al. [118], who used a mathematical approach to elucidate the relationship between the porosity and the solvent diffusivity. Another approach of activating cellulose is its heat activation in DMAc at a high temperature [115,120]. The activation of cellulose by heating in DMAc (150 °C, close to solvent boiling point) is a very common technique [120,121,122]. At high temperatures, the solvent diffuses into the fiber and the solvent vapors cause fiber swelling [117]. This one-step strategy is more advantageous than solvent-exchange activation because it allows for the easy handing of large amounts of cellulose. However, the elevated temperature might initiate cellulose degradation [104]. The mechanical pretreatment of cellulose, such as high-speed stirring, causes its degradation during dissolution [123]. Multiple studies, however, have shown that cellulose degradation during mechanical treatments is not significant [67,115].

**Table 2:** Representative examples of cellulose dissolution in DMAc/LiCl, including pretreatment strategies or high-temperature dissolution.

Cellulose Source	Cellulose DP	Pretreatment	Dissolution Conditions			Ref.
			Cellulose Concentration (wt.%)	Temperature (°C)	Time (h)	
Wood pulp	~800	Solvent exchange with DMAc	0.4	40	24	[124]
Cotton	~5350	Freeze-drying	3.0–5.0	80	74.5	[105]
Cotton	~2850	Solvent exchange with methanol	6.0–15.0	RT	24–48	[97]
Cotton	4580	High-temperature dissolution	2.2	150	2.5	[125]
Cotton	~11,300	Heat activation	1.2	150	48	[120]
Cotton	~1100	Heat activation	1.0	100	50	[122]
Cotton	~21,000	Heat activation	1.5	150	24–48	[121]
Cotton	~10,900	High-temperature dissolution	1.0	150	52	[126]
MCC	ND	Freeze-drying	5.0	80	24	[8]

### 4.3. N-Methylmorpholine-N-Oxide (NMMO)

NMMO stands out as the superior cellulose solvent among amine oxides, and it constitutes another important non-derivatizing cellulose solvent due to its ease of preparation and broad application range [36]. Usually, cellulose can be directly dissolved in NMMO monohydrate containing ~13% water at concentrations as high as 23% at temperatures between 80 and 120 °C [1,127]. The importance of NNMO can be exemplified by the lyocell process, the second most-used industrial cellulose dissolution process after the viscose process. The ability of NNMO to dissolve cellulose without the need for prior activation/pretreatment and derivatization allows the lyocell process to significantly shorten the production route in comparison to the viscose process. Since the release of highly toxic CS_2_ is one of the major environmental concerns in the viscose process, the lyocell process is considered to be an environmentally friendly process for the industrial production of textile fibers [128]. Additionally, the recovery of NMMO in the lyocell process exceeds 99% [1,91]. Lyocell fiber has superior properties over viscose rayon in many aspects such as strength (in both wet and dry states), wettability, and elasticity. The final textile fibers are also shinny and pleasant to touch [129].

The pre-swelling of cellulose in NNMO is required in order to obtain homogenous solutions. It is typically achieved by dispersing cellulose fibers (10–15%) in a dilute aqueous solution of NMMO: (60% NMMO and 20–30% water) [130]. Under these conditions with high water contents (>15%), NMMO does not dissolve cellulose [128]. In order to achieve dissolution, water is gradually evaporated under vacuum at an elevated temperature. This makes the process somewhat energy-intensive and lengthy [131]. Even though the lyocell process is considered to be a superior industrial method to produce cellulose fibers compared to the viscose process [100,128], it has many disadvantages such as solvent and polymer oxidation and rather high temperatures [1,100,130].

**Figure 5:**
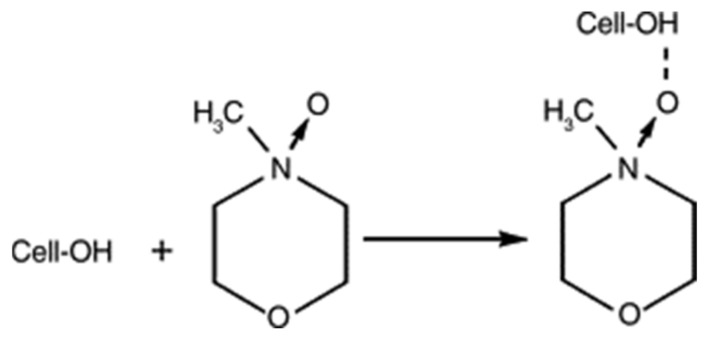
Typical mechanisms of cellulose dissolution in NMMO. Reprinted from [134] with permission from Elsevier © 2021.

Mechanistically, the dissolution of cellulose in NMMO is attributed to the strongly dipolar nature of the active NO moiety. The interaction of oxygen of the NO group with cellulose occurs via the formation of one or two hydrogen bonds with an anhydroglucose unit and leads to the disruption of the native intra- and intermolecular hydrogen bonds of cellulose. This ultimately results in the formation of a soluble hydrogen-bonded complex between the cellulose and NMMO [36,132]. The absence of water is very crucial in maintaining the cellulose-dissolving ability of the NMMO system. When the molar ratio of water to amine oxide reaches 2, the cellulose-dissolving ability of NNMO abruptly decreases because water coordinates with the oxygen atom of NMMO, thus precluding the formation of hydrogen bonds with cellulose [133]. Figure 5 illustrates the typical mechanism of cellulose dissolution in NMMO.

#### Strategies for Improvement of Cellulose Dissolution in NMMO

To overcome the disadvantages of the lyocell process, several strategies have been proposed. NMMO is prone to thermal degradation due to homolytic and heterolytic cleavages at temperatures typically employed during cellulose dissolution and/or solvent recycling processes. The degradation products of NMMO include N-methyl morpholine, morpholine, formaldehyde, and few unstable reactive species such as N-methylmorpholiniumyl radical and *N*-(methylene)morpholinium cation [128,135]. This not only results in the loss of the solvent but also causes cellulose degradation and negatively impacts product performance. To avoid solvent degradation, it is usually stabilized via the addition of alkali and radical scavengers (e.g., NaOH and propyl gallate (PG)) [136]. Rosenau and coworkers [135] reported that PG and 3-oxachromanol compound 2,4,5,7,8-pentamethyl-4Hbenzo[1,3]dioxin-6-ol (PBD) were found to be efficient stabilizers that worked by trapping and/or scavenging the solvent degradation products. In a later study, Wendler et al. [136] reported a novel polymeric stabilizer system consisting of iminodiacetic acid sodium salt (ISDB) and benzylamine (BSDB), each covalently linked to a styrene/divinyl benzene copolymer. This novel polymeric stabilizer system was found to be more effective in stabilizing the cellulose/NMMO solution in the presence of additives possessing carboxyl groups, or surface-active additives, compared to the NaOH/PG stabilizer.

Dogan and Hilmioglu [137] demonstrated that the use of microwave heating significantly shortened cellulose dissolution time and lowered the energy consumption compared to the conventional dissolution process. The authors concluded that microwave heating with a power of 210 W at a frequency of 2450 MHz provided the best results. Sayyed et al. [138] reported that the ultrasound treatment of a mixture of wood pulp in NNMO/water resulted in enhanced polymer swelling at a shorter time. The researchers also demonstrated that the ultrasound pretreatment of cellulose with an ultrasound frequency of 37 kHz and a power of 320 W significantly reduced the particle size of cellulose in the slurry and decreased the time and temperature needed for the dissolution without impacting the solution quality.

### 4.4. Ionic Liquids (ILs)

Ionic liquids (ILs) are molten salts that remain liquids at or below 100 °C [139]. ILs mostly have low vapor pressure as a result of the strong ionic interaction between the constituent ions and thus do not emit potentially hazardous vapors during operation and handling. They exhibit high thermal stability and (generally) non-flammability, although few have been used as hypergolic fuels [140,141,142,143]. However, because the ILs used for cellulose dissolution are highly hydrophilic and thus absorb water, they are more difficult to recycle than conventional solvents [144]. There are many overgeneralizations that hang over the field such as the idea that ILs are non-toxic and green. In the long run, however, one has to be careful not to assign certain traits to all ILs since they are different classes of chemicals, and many quite exotic ILs have been prepared, including polymeric ILs [145] and double salt ionic liquids (DSILs [146]). ILs show the remarkable ability to dissolve or disperse numerous organic and inorganic compounds [147].

The first report of molten salts was introduced in the early 1900s by Walden [148], and a publication in C&EN in 1997 launched a renaissance of the field [149]. Since then, numerous ILs have been synthesized with a broad range of cation–anion combinations and a diverse suite of physicochemical properties, behaviors, and solvation abilities [142]. Along with the development of new ILs and their commercial availability, the proper understanding of their properties followed and fascinating properties of these salts inspired many researchers, especially those concerned with potential applications in greener technologies, to understand their true potential and behavior.

The discovery of dissolution and deconstruction of cellulosic feedstock in ILs was made by Rogers in 2002; this work led to the use of ILs as solvents for cellulose and other biopolymers [150]. In 2007, the Rogers group followed this work with the first report that ILs could dissolve recalcitrant lignocellulosic biomass such as wood [151]. In 2009, it was found that cellulose-rich material can be obtained in a single step via the complete dissolution of softwood and hardwood in the ionic liquid 1-ethyl-3-methylimidazolium acetate followed by separation [152]. Currently, there are multiple ILs for the dissolution, pretreatment, and/or processing of biopolymers and biomasses (chitin/chitosan [153], silk fibroin [154], lignin [155], starch [156], keratin [157], etc.). The most efficient anions used in ILs for cellulose dissolution are basic ones such as chloride (Cl^−^), carboxylates (e.g., acetate ([OAc]^−^), formate ([HCOO]^−^), and propionate ([OPr]^−^)) or derivatives of mineral acids (e.g., diethylphosphate ([(EtO)_2_PO_2_]^−^) and dimethylphosphate ([(MeO)_2_PO_2_]^−^) [158]). Regarding the cations, effective cellulose dissolution has been shown in imidazolium-based, pyridinium-based, ammonium-based, and phosphonium-based ILs [147], although the ILs with unsaturated heterocyclic cations (imidazolium and pyridinium) are the most successful ones in cellulose dissolution [147,159]. Among those, the most efficient imidazolium cations include 1-butyl-3-methylimidazolium ([C_4_mim]^+^]), 1-ethyl-3-methylimidazolium ([C_2_mim]^+^), 1-allyl-3-methylimidazolium ([Amim]^+^), and 1,3-dimethylimidazolium ([C_1_mim]^+^) [147,159], while pyridinium cations include 1-propyl-3-methylpyridinium ([C_3_MPy]^+^), 1-butyl-3-methylpyridinium ([C_4_MPy]^+^), and 1-pentyl-3-methylpyridinium ([C_5_MPy]^+^) [160]. Somewhat more unusual cations have also been used, and commercialization efforts are ongoing for “superbase-based” 7-methyl-1,5,7-triazabicyclo[4.4.0]dec-5-enium acetate ([mTBDH][OAc]) and 1,5-diaza-bicyclo[4.3.0]non-5-enium acetate ([DBNH][OAc]) in Aalto, Finland [161].

Cellulose, extracted from different sources, has been successfully dissolved in various ILs [40,150,162,163,164] (representative examples are presented in Table 1; however, the area is quite broad and only few representative examples have been covered [147]). Depending on the cellulosic feedstock, the molecular and structural properties of cellulose, and the dissolution power of the selected ILs, the need and conditions of pretreatment and the dissolution conditions (e.g., cellulose load, heating rate, and process duration) have to be optimized. In general, dried cellulose samples are added to ILs and thermally heated with a heating plate [150], oil bath [165,166], or by means of a microwave oven [40,150,162,167]. Although many of the ILs are liquids, if/when solids (e.g., 1-butyl-3-methylimmmidazolium chloride), they are preheated above their melting point to make them fluid enough to mix with cellulose. Usually, the solutions are protected from air to prevent water adsorption because many ILs are hydrophilic and tend to accumulate water from air; ~3–7% water in an IL makes it ineffective for cellulose dissolution. The solutions are subsequently heated using thermal heating such as an oil bath for 1–24, or by applying short microwave pulses of 3–5 s for several minutes [40,150,162,168]. The overheating is prevented by vortexing the solution between microwave pulses [150] because the excessive heating of the cellulose IL mixture induces cellulose pyrolysis [150]; the longevity of microwave pulses should ensure that the sample is not overheated during dissolution [167].

The source of cellulose and thus the structural characteristics of cellulose polymer have also been extensively studied. As such, Swatloski et al. successfully dissolved 5–10% of relatively low-molecular-weight cellulose (e.g., cellulose-dissolving pulps with DP ~1000, fibrous cellulose, and filter paper) in imidazolium-based ILs [150]. Heinze et al. dissolved a very high concentration of cellulose (1–36%) with a DP that ranged from 290 to 1200 in several ILs [163]. Dissanayake et al. successfully dissolved 5% cotton fibers that are well known for their high molecular weight (DP ~7000–8000) and high crystallinity (~75–80%) in numerous imidazolium-based ILs [40,162].

There are multiple reviews available for understanding a mechanism of cellulose dissolution in ILs [169,170]. Swatloski et al. first suggested that the anions interact with the hydroxyl groups of cellulose molecules and disrupt extensive intra- and intermolecular hydrogen bonds [150]. Because the anion concentration is high in ILs, the use of ILs results in the dissolution of cellulose at a much higher load compared to the DMAc/LiCl solvent system [150]. Later, several experiments and simulation studies showed that the anions and –OH groups of cellulose form hydrogen bonds during the dissolution process [171,172]. Currently, it is well-established that the anions of ILs play the most important role in cellulose dissolution [159,171,173], and solubility seems to follow their basicity [147]. Specifically, Zhao et al. noted that the dissolution power of ILs increases when the anion has a hydrogen-bonding acceptor with a high electron density (anion–cellulose interaction changes in descending order Cl^−^ > [CH_3_COO]^−^ > [(CH_3_O)_2_PO_2_]^−^ > [SCN]^−^ > [PF_6_]^−^), shorter alkyl chains, and no electron-withdrawing groups [171]. For a quantitative comparison between ILs capable to dissolve the polymer, empirical solvent descriptors *α*- and *β*- (*α—*hydrogen bond acidity [174]; *β—*hydrogen bond basicity [175,176]) elucidated from Kamlet–Taft equation are used. Here, the *β*-parameter is determined by the basicity of the IL anion, while α-parameter is elucidated from IL “as a unity”, so ILs with the same cation but different anions demonstrate different *α*-values. It was found that high *β*-values (*β* > 0.8) are required for cellulose dissolution to take place [132,166,177].

**Figure 6:**
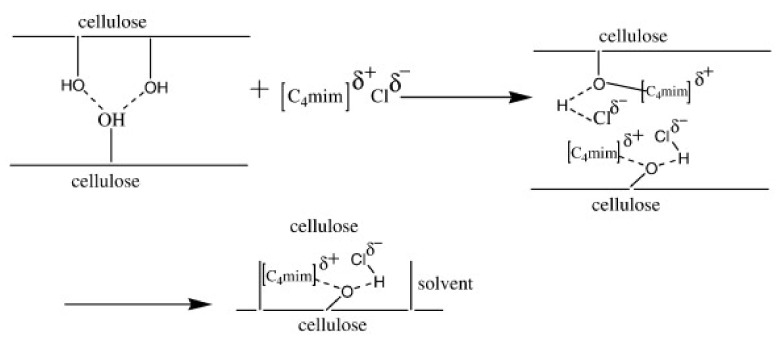
Typical mechanism of cellulose dissolution in IL. Reprinted from [180] with permission from Elsevier © 2021.

The dissolution studies conducted with numerous ILs with the same anion but different cations made it clear that the cations also play an important role in cellulose dissolution [163]. Later studies confirmed that while both anions and cations play crucial roles in cellulose dissolution [178,179], the role of cations is secondary to that of anions [158,173]. The exact mechanism of how cations are involved in cellulose dissolution is still under discussion [147,158,159]. Zhang et al. proposed that cations also make hydrogen bonds with cellulose [178], and Li et al. suggested that van der Waals interactions between cations and cellulose molecules play the important role in formation of these hydrogen bonds [159]. As discussed by Zhao et al., both the type of anion (e.g., imidazolium, pyridinium) and the alkyl chain lengths are important for dissolution [171]. A typical mechanism of cellulose dissolution in ILs is presented in Figure 6.

#### Strategies for Improvement of Dissolution of Cellulose in IL-Based Systems

Despite numerous beneficial properties that led to their widespread applications in many fields, the typical problems associated with ILs are their highly hygroscopic nature and (for some) high viscosity that impairs cellulose dissolution [164]. First, the presence of water hinders cellulose dissolution in ILs due to competitive hydrogen bonding with cellulose [150]. Therefore, the proper dehydration of cellulose and ILs is an important step. The need for the proper dehydration of ILs for reuse is the most complex technological hurdle to overcome for scaling up.

Increasing the temperature of cellulose–IL mixtures facilitates dissolution and reduces dissolution time [164], but excessive heating increases the chance of cellulose degradation [167]. As insights into the interaction between ILs and cellulose become deeper, further discoveries regarding pretreatments and cosolvents have continued apace to facilitate cellulose dissolution in ILs. Adding an aprotic cosolvent, such as dimethylsulfoxide (DMSO), *N*,*N*-dimethylformamide (DMF), and DMAc has been found to enhance the dissolution power of ILs by decreasing dissolution time and temperature [162,164,166,181,182]. As discussed by Li et al., there are different theories regarding how cosolvents facilitate cellulose dissolution [158]. Some researchers have speculated that the addition of cosolvents dissociates ILs into solvated cations and thus liberates anions [164,182], which increases anion–cellulose interactions and promotes dissolution. Others have suggested that cosolvents improve the mass transport properties of the solution (decrease viscosity and increase ionic conductivity) and facilitate dissolution [162,183,184]. As discussed by Wang et al., the high viscosity of some ILs is thought to contribute to difficulties in cellulose dissolution, and less viscous ILs result in better transport, are easy to handle, and disperse cellulose well [147].

As of yet, no ideal conditions or inexpensive ILs have been identified for cellulose dissolution for commercial-scale applications, although the technology has huge potential. Indeed, attempts to commercialize cellulose processing in ILs have already been done by BASF, but these were not successful due to the need to recycle the currently expensive ILs [185]. Funded by U.S. Department of Energy, Joint BioEnergy Institute (JBEI) worked for years on ILs that separate cellulose from lignin for sugar production [186]. Finnish Metsä Fibre is working on developing textile fibers from softwood pulp using ILs.

According to the current volume of research and their proven cellulose dissolution capacity, ILs will soon undoubtedly become the best replacement for common solvents used for cellulose dissolution in a much easier and environmentally friendly way compared to currently available technologies. Some representative examples are presented in Table 3, although it is worth noticing that most of the dissolution studies lack specific information to distinguish between the dissolution and degradation of cellulose.

## 5. Cellulose Regeneration and Shaping

While many scientists were interested in understanding the mechanism of cellulose dissolution in various solvents, others sought further advances in converting these solutions into usable products. In many studies, cellulose solutions have been shaped, chemically modified, or depolymerized to produce multipurpose materials (bioplastics), cellulose derivatives, and cellulosic fuels, respectively [143].

Among these directions, the production of cellulose bioplastics is a highly active and promising area of research with a recent surge in interest due to the environmental pollution caused by non-degradable petroleum-based products. During the production of various materials, the cellulose solution is shaped into a desired form (e.g., filaments, films, and beads) via various processes (dry- and wet-jet spinning, electrospinning, casting, etc.) and then added to an anti-solvent such as water and alcohol (this step is called coagulation), and cellulose is recovered in a desired form. During coagulation, protic solvents compete with the cellulose–solvent interactions and promote cellulose precipitation. In this manner, many bioproducts can be manufactured from cellulose and its composites with other biopolymers. On the other hand, the abundance of free hydroxyl groups in regenerated cellulose material opens avenues for the further derivatization of the prepared materials. Herein, we review the fundamental aspects of processing cellulose solutions into various products with an emphasis on preparations and applications.

### 5.1. Cellulose Hydrogels

Hydrogels are three-dimensional polymer networks that can absorb, retain, and release water in a reversible manner [193]. They can be prepared by the gelation of cellulose solutions. Since hydrogels made from cellulose or its derivatives are not always mechanically strong, crosslinkers (e.g., epichlorohydrin (ECH), aldehydes, and urea derivatives) [193] could be introduced to impart strength into materials. Thus, Chang et al. prepared superabsorbent hydrogels from cellulose, carboxymethylcellulose (CMC), and ECH by gelating the cellulose solution at 60 °C for 12 h [194]. These hydrogels showed a remarkable swelling ratio of 1000 in water and demonstrated smart swelling/shrinking behavior in aqueous salt solutions. In another study, cellulose-based gels were made by heating a cellulose solution with added ECH at 50 °C for 20 h or by freezing the solution at −20 °C for 20 h [195]. Kadokawa et al. sandwiched a cellulose solution in [C_4_mim]Cl IL between glass plates and gelated the solution for 7 days at ambient temperature [187], and a transparent gel was prepared by regeneration in ethanol.

The surface polarity of cellulose gels is largely impacted by the type of coagulant. Thus, Isobe et al. cast 1 mm thick layers of cellulose solutions on glass plates and immersed them into alcohol and aqueous coagulation baths for 1 h [196]. Wang et al. also cast 1 mm thick layers of cellulose solutions onto glass support, immersed them into ethanol for gelation, and washed them with ethanol and water to prepare a translucent gel [197]. Interestingly, instead of simple gelation, cellulose-based hydrogels can also be made by exploiting irradiation-induced crosslinking (γ-radiation) or crosslinking and grafting followed by washing with DI water [198,199,200].

Cellulose-based hydrogels have widespread applications due to their hydrophilicity, biocompatibility, stimuli-sensitivity, and exceptional adsorption capability (high swelling ratio). Blending cellulose with other biodegradable polymers, such as chitin or alginate, combines the characteristics of cellulose and other polymer and produces multifunctional composite hydrogels for specific applications [201]. Thus, cellulose composite hydrogels have been prepared for heavy-metal adsorption (e.g., Cu II, Cr, and Pb) [199,202], control adsorption [203,204], and dye adsorption [200], as well as for other applications where their swelling capacity [205], deformation recovery, and strength [206] were important. Furthermore, additional functionalities have been introduced to cellulose hydrogels by grafting new functional moieties onto cellulosic surfaces (e.g., anion-exchanger [207]). Cellulose composite hydrogels are promising materials for a diverse range of fields, including biomedical, pharmaceutical, water purification, and wastewater treatment applications.

### 5.2. Aerocelluloses

Aerogels are unique materials with high specific surface area, low density, and low thermal conductivity [208]. They show great promise as sorbent materials, insulators, and materials for separation in chromatography columns and commercial extractors [209,210]. Initial attempts to produce cellulose aerogels (so-called aerocellulose monoliths) were reported as early as the 1930s [211]. Aerogels are prepared by replacing the area occupied by a liquid in a cellulose hydrogel (see previous section of this review) by air [212]. During this process, the overall volume and the network structure of the gel remain largely unchanged. Currently, there are a large number of ongoing studies on cellulose aerogels with very high specific surface area and improved pore characteristics.

Cellulose aerogels are usually prepared via the supercritical CO_2_-drying of regenerated-cellulose-based hydrogels [210,212,213]. In brief, a cellulose solution is poured into a mold and heated at 50 °C until a gel is formed (different gelation times are required for different solvents). Then, cellulose gels are regenerated in water, neutralized (if needed), solvent-exchanged with anhydrous acetone or ethanol, and subsequently supercritically dried. The resultant aerocellulosic monoliths possess a continuous interconnected network of pores. Similar to cellulose-based hydrogels, crosslinkers are used when aerogels are not mechanically strong [212]. The specific surface area of aerocellulosic materials ranges from 100 to 400 m^2^/g [210,213,214,215]. Instead of supercritical drying, freeze-drying can be employed to produce cryogels that have also been shown to have high surface area and porosity. For example, Wang et al. prepared highly porous aerogels via the freeze-drying of cellulose gels (surface area = 190–213 m^2^/g; pore size = 10–60 nm) [197]. It has been shown that various additives (inorganic salts, surfactants, porogens, etc.) can affect the specific surface area and porosity of aerocellulose monoliths [213,214,216].

Cellulose aerogels have widespread applications due to their high specific surface area, interconnected pore structure, light weight, and tunable properties coupled with the inherent beneficial properties of cellulose. They have been functionalized by surface coating, surface grafting, and the incorporation of active molecular or atomic species (graphene and actives of various types) to diversify their applications. For instance, cellulose aerogel monoliths have been functionalized for tunable oleophobicity [208], the efficient removal of heavy metals [217] and dyes [210,218,219], and improved antimicrobial [220] and antioxidant activities [219].

### 5.3. Cellulose Membranes

Films and membranes prepared from petrochemical compounds have widespread applications ranging from simple packaging materials to ultrafiltration devices and smart materials. However, there is an increasing trend of producing films and membranes from cellulose as alternatives to non-biodegradable synthetic membranes. Several methods for converting cellulose solutions into films have been reported. Many studies have reported the preparation of cellulose films by casting cellulose solutions on glass substrates followed by (optional) gelation and immersion in anti-solvents to make hydrogels, which were later vacuum- or air-dried [167,221,222,223,224,225]. The type of coagulant and the temperature of the coagulation bath influence the material characteristics of cellulose films [222,223,225].

Garnier et al. prepared films on glass slides covered with cellulose acetate solutions followed by air-drying, regeneration, washing, and re-drying [226]. Da Róz et al. prepared cellulose films by spin-coating a cellulose solution onto a glass substrate followed by vacuum-drying and washing with DI water [227]. Zhang et al. precoagulated cellulose solutions on glass plates, coagulated the films in an H_2_SO_4_ aqueous solution, washed them with water, and air-dried them [228]. Ma et al. and Acharya et al. cast cellulose solutions on glass supports and gelated the solution at ambient conditions prior to regeneration and drying [8,39,191]. Wang et al. introduced hot-pressing to transform the regenerated cellulose hydrogels into uniform, transparent, and flexible films with better tensile properties compared to films prepared by regular drying at 25 °C [229]. The plasticization of cellulose hydrogels with, e.g., glycerol or polyethylene glycols (PEGs), typically improves the tensile strength and elongation at break of dried films [228].

Many studies have reported on the preparation of multifunctional cellulose films for various applications. These films have also been functionalized through surface modification or blending with active molecules (composite films) for specific applications. Specifically, films with improved tensile strength [191,230], antibacterial activity [224,227], and grafted moieties for heavy-metal adsorption [231] have been prepared through functionalization. Accordingly, Turner et al. incorporated active enzymatic species to produce biologically active cellulose membranes [167]. Liew et al. and Sadasivuni et al. prepared composite films for supercapacitors and flexible electronics [232,233]. Sievens-Figueroa et al. prepared water-soluble hydroxypropyl methyl cellulose films containing drug nanoparticles for pharmaceutical applications [234]. Several other studies have focused on producing porous cellulose films for ultra and microfiltration [221,222]. Flexible, transparent, and biodegradable cellulose-based films with excellent mechanical properties have been utilized as packaging materials with certain oxygen- and water-permeation properties [223,235]. Cellulose films are also used for decontamination, purification, filtration, separation, and electronic applications [168,221,222,231,232,233,236].

### 5.4. Cellulose Fibrous Materials

Cellulose fibers can be prepared by the dry- or wet-jet spinning [190,237,238,239] and electrospinning [189,240,241] of cellulose solutions and their derivatives. For instance, in electrospinning, cellulose solution derivatives in volatile solvents are electrospun onto stainless steel or aluminum foils mounted on a rotatable drum, whereas solutions of cellulose in non-volatile ILs are electrospun into coagulation baths (for non-volatile ILs) [237]. In general, an electrospinning system consists of a high-voltage power source that is connected to both a solution-supplying system and a grounded electrode, the spinneret, and a collector (rotating drum for volatile solvents (VOCs) or coagulation bath for ILs [189,242,243,244,245]. Electrospinning is conducted either under pressure or in a “free-fall” manner using 15–40 kV voltage; some systems combine voltage with centrifugal forces. In case of a drum collector, the electrospun fibers are collected while the solvent evaporates. In case of a coagulation bath, electrospinning is conducted into anti-solvent coagulation baths [237,238], and then the wet cellulose nanomat is washed and subsequently dried.

Many parameters, including solution viscosity, flow rate, the size of the air gap between ejectors (needles) and the collector, coagulation medium (if required) and its temperature, and filament tension influence the material characteristics of the final product [189,237,238]. In this manner, cellulose and its derivatives dissolved in different solvent systems have been transformed into nanofibrous materials using various cellulose sources such as natural wood flour from pine wood [246], hemp cellulose [247,248], cotton linters [249]. For instance, Kosan et al. prepared cellulose fibers with 13.2–19.6% cellulose dissolved in different ionic liquids [237]. Interestingly, the fiber properties were largely influenced by the type of IL, and fibers made from the ILs with a basic Cl^−^ anion had a higher fiber tenacity and lower elongation compared to fibers made from acetate containing ILs. Quan et al. also electrospun cellulose IL solutions and studied the effect of solution viscosity on fiber properties [189]. The authors showed that the 4% cellulose solution gave the best fiber quality, while high viscosity prevented smooth solution flow. Kim et al. produced hollow fibers from cellulose dissolved in IL [192]. Frenot et al. electrospun fibers from cellulose and its derivatives dissolved in different solvent systems at various concentrations [240]. The authors experienced difficulties in electrospinning cellulose dissolved in the DMAc/LiCl solvent system due to the presence of high amounts of salts and the nonvolatility of the solvent. Zhang et al. were able to incorporate carbon nanotubes into a cellulose solution prior to electrospinning and showed that cellulose–carbon nanotube composite fibers possessed improved mechanical properties and thermal stability compared to those made from neat cellulose [190].

It has been reported that regenerated cellulose fibers/filaments are expected to be used in the textile industry [237] as a simple and environmentally safe alternative for the viscose process [239] or in applications where the surface area is important because the nanomaterials prepared from cellulose had a fiber diameter in the nm range. Regenerated cellulose filaments have shown good flexibility, silk-like luster, and excellent dye exhaustion [239]. The materials have applicability in ultrafiltration, electronics, tissue engineering [189], and drug delivery applications because they could be tuned for drug release rate [240].

## 6. Potential Impacts: Application of Cellulose Materials

### 6.1. Biomedical Applications

Regenerated cellulose materials are increasingly used for biomedical applications in, e.g., implants, wound dressings, and scaffolds. They possess many properties favorable for tissue engineering and regenerative medicine, including hydrophilicity, water and O_2_ permeability, biocompatibility, stimuli-sensitivity, and non-toxicity. They can be prepared as three-dimensional structures with tunable properties for targeted applications, and there are existing production methods available for producing 3D cellulose scaffolds including molding. However, the possible applications of cellulose-based products are basically determined by their inner structural morphology (porosity and pore size), physical properties, and uniformity for improved cell interactions and guided cell proliferation [250].

Many studies have reported on the use of cellulose-based hydrogels for wound-healing applications. Mao et al. showed that carboxymethylcellulose (CMC) hybrid hydrogels containing antimicrobial agents based on Ag and ZnO had a better antimicrobial activity and accelerated wound healing compared to neat hydrogels [251]. Loh et al. showed that bacterial–cellulose-based hydrogels can be seeded with epidermal keratinocytes and dermal fibroblast cells for improved wound healing [252]. Zander et al. developed nanocellulose hydrogels crosslinked with metal cations (Ca^2+^ and Fe^3+^) and surface-modified these hydrogels with an extracellular matrix protein fibronectin [253]. The authors showed that cell adhesion was significantly improved due to surface modification and suggested the use of these materials as wound-healing dressings.

In regenerative medicine, damaged soft and hard tissues are replaced or restored with three-dimensional scaffolds to support cell attachment, survival, and proliferation that assist cell organization and tissue repair. Cellulose-based scaffolds mimic natural extracellular matrices, and they have been used in the regenerative treatments of many tissues (heart, bone, cartilage, blood vessels, and liver) [254]. Liu et al. showed that three-dimensional cellulose films and aerogels (scaffolds) aided human tumor cell attachment, survival, and proliferation [255]. These scaffolds could be suitable for the development of in vitro disease models to study tumor development.

In addition, cellulose hydrogels have remarkable strength and flexibility that make them ideal candidates as load-bearing soft tissues or cartilage replacements [256,257]. It was found that chondrocytes isolated from the knee joints of bovine calves exhibited excellent adhesion and proliferated on activated cellulose scaffolds [256]. Mathew et al. showed that the partially dissolved cellulose nanocomposite scaffolds could be used in ligament or tendon substitution [258]. The authors reported that the dissolved celluloses act as extracellular matrices of tissues while the undissolved cellulose particles reinforce the scaffold. Those hydrogels were found to be stable against cyclic loading and unloading and were supportive for human ligament and endothelial cell adhesion and proliferation.

3D bioprinting is an emerging technique for fabricating patient-specific tissue structures from cellulose-based materials with high accuracy, repeatability, and customized pore characteristics [259,260,261]. Cellulose 3D bioprinting, when implemented across the board, could facilitate the large-scale production of biomaterials with high specificity. However, even though the production of cellulose-based organs or tissues for in vivo applications is fascinating and rewarding, it is extremely challenging due to the complexity of human anatomy and physiology. Because 3D printing technique can produce tailored hydrogel structures, it will revolutionize patient-specific tissue engineering at some point in the future.

### 6.2. Sorption Applications

Regenerated cellulose materials hold great potential for absorbent applications. The combination of excellent physical properties such as high porosity and surface area [213], lower crystallinity, ease of functionalization and composite development, non-toxic nature, and abundancy make them ideal absorbents for dyes, heavy metal ions, oils, and CO_2_, among others. Dassanayake et al. used aerocelluloses with activated carbon for CO_2_ adsorption [262], and the authors reported 5.8 mmol·g^−1^ of CO_2_ adsorption at 0 °C and 1 atm and 3.7 mmol·g^−1^ of CO_2_ adsorption at 25 °C and 1.2 atm, attributed to the microporous structure and high surface area of the activated carbon. Nguyen et al. reported methyltrimethoxysilane (MTMS)-functionalized cellulose aerogel for crude oil adsorption [263], and the authors achieved 18.4, 18.5, and 20.5 g/g of absorption of RB, Te Giac, and Rang Dong oils at 25 °C, respectively. The differences in absorption were attributed to the difference in the viscosity of the oils, where oil with lower viscosity more efficiently penetrated the porous network than the oil with higher viscosity. The highest absorption of 24.4 g/g was achieved for RB crude oil at 40 °C. Similarly, Wang et al. studied the oil–water separation using porous and hydrophobic silanized cellulose prepared via a reaction of microcrystalline cellulose (MCC) with hexadecyltrimethoxysilane (HDTMS). They obtained 99.93% separation efficiency towards a vegetable oil–water mixture, which only insignificantly dropped to 99.77% after being recycled ten times [264].

Zhan et al. used a polyethyleneimine-modified crosslinked cellulose–sodium alginate composite for the adsorption of Cu(II), Zn(II), and Pb(II) in aqueous solutions [265]. The authors reported maximum adsorption values of 177.1, 110.2, and 234.2 mg·g^−1^ for Cu(II), Zn(II), and Pb(II) ions, respectively, which was attributed to the presence of chelating carboxylate and amino groups along with the high porosity of absorbents. Similarly, the heavy metal ion adsorption properties of cellulose–chitosan composites, prepared by co-dissolving cellulose and chitosan in [C_4_mim]Cl IL, were studied by Sun et al., and adsorption capacities of 0.417, 0.303, 0.251, 0.225, and 0.127 mmol·g^−1^ were achieved for Cu(II), Zn(II), Cr(VI), Ni(II), and Pb(II), respectively [266].

Dye adsorption from wastewater is an extensively studied application of regenerated cellulose materials because they offer inexpensive, reusable, and energy-efficient remediation. Ren et al. prepared regenerated-cellulose–graphene oxide composite aerogels via solution mixing-regeneration and freeze-drying process for the high-efficiency adsorption of methylene blue [267]. The authors reported a 99% removal efficiency of methylene blue (MB) with the addition of 0.5% graphene oxide. Moreover, the dye-removal efficiency dropped to 90.5% even after five cycles of reuse. The adsorption was driven by the electrostatic interaction and facilitated by the 3D network structure and high specific surface area of the adsorbent. Salama [268] prepared a novel biocomposite from cellulose, silk fibroin, and calcium phosphate for the high-efficiency removal of MB. The author reported that the cellulose/silk fibroin biocomposite exhibited a higher removal efficiency of 172.4 mg·g^−1^ for MB than the cellulose/silk fibroin blend (120.4 mg·g^−1^). A higher adsorption was attributed to the presence of additional chelating binding sites created by the presence of hydroxyapatite. Extensive research efforts on the optimization of existing technologies utilizing the intriguing properties of cellulose will help widen the scope of its applications and maintain its use as a promising material for industrial application.

### 6.3. Energy Applications

In the quest to find inexpensive, eco-friendly, and renewable energy generation and storage systems including photovoltaics and supercapacitors, regenerated cellulose has gained increasing attention [269,270,271,272,273]. Particularly, the use of cellulose in the field of supercapacitors has garnered significant research interest for the preparation of supercapacitors with long life cycles, high-power densities, and the rapid propagation of charge/discharge [274,275,276,277,278]. These supercapacitors have found their uses in different applications, such as computer memories, hybrid electric vehicles, power-driven tools, and consumer industrial power management [279].

Zhao et al. demonstrated an effective strategy for preparing highly flexible and conductive cellulose films for electrodes in supercapacitors. These films were cellulose/poly(3,4-ethylenedioxythiophene) PEDOT: poly(styrene sulfonate) (PSS) composites, with incorporated multiwalled carbon nanotubes (MWCNTs) [272], and they were prepared using [C_4_mim]Cl IL. For this, cellulose and 3,4-ethylenedioxythiophene (EDOT) were co-dissolved in IL while forming supramolecular self-assembly. Then, MWCNTs were dispersed in the resulting solution, and the solution was cast onto silicon wafers. The composites were then regenerated through immersion into distilled water to produce composite hydrogels. The obtained hydrogels were impregnated with aqueous solutions of PSS, and then EDOT in the matrix was polymerized by the addition of ammonium persulfate (APS) polymerization initiator. Following EDOT polymerization, the hydrogels were dried at 60 °C. The films were used to construct electrodes, and these electrodes showed good capacitive properties (380 F·g^−1^), a fast charge−discharge ability, and a maximum energy density of 13.2 Wh·kg^−1^.

In another study, Liu et al. utilized a template polarization method to prepare a flexible conductive composite from a cellulose scaffold via the polymerizing pyrrole (Py) monomer present throughout [273]. Such polymerization results in interactions between the hydroxyl groups of cellulose and amine groups of pyrrole [273]. The supercapacitor made out of this electroactive composite exhibited a specific capacitance of about 308–392 F·g^−1^ at current densities of 0.1–0.4 A·g^−1^, with 82% capacitance retention after 1000 charge–discharge cycles. The porosity of regenerated cellulose substrate could also be valuable for the even distribution of other conductive polymers such as polyaniline, and the in situ polymerization of aniline has also been achieved. Thus, Liu et al. conducted the in situ polymerization of aniline inside a regenerated cellulose scaffold [129], and the obtained flexible film exhibited considerable conductivity, although its specific capacitance was relatively low at only 160 F·g^−1^ [129].

Lithium-ion batteries (LIBs), with their high power, high energy density, and long cycle life, are highly demanded in wearable and portable electronic devices [280,281,282]. However, they have safety issues related to the low thermal stability of the polyolefin-based commercial separators and the leakage of liquid electrolytes at elevated temperatures [283]. The potential applications of cellulose and cellulosic materials could also include electrodes and matrices for electrolytes [284].In addition, due to their high chemical and thermal stability, they could be good alternatives to polyolefin separators in LIBs [1,285]. Cellulose derivatives, such as methyl cellulose, carboxymethyl cellulose, and cyanoethylated cellulose, are mostly used to prepare microporous membranes for gel polymer electrolytes [285,286,287].

There have been very few research studies on the direct use of cellulose or regenerated cellulose in LIBs [282,288,289]. Wan’s group introduced cellulose aerogel membranes (CAMs) [282]. The CAM was used as a matrix for gel polymer electrolyte in LIBs. The highly porous CAMs with numerous –OH groups can absorb liquid electrolytes very quickly, resulting in CAMs with high ionic conductivity. The LIBs with gelled CAMs exhibited excellent electrochemical performance. Due to the intrinsic high thermal stability of cellulose, LIBs were found to work stably and safely even at 120 °C. Wang et al. developed a lightweight, flexible, and foldable conductive film comprising cellulose paper fused with nanoparticles of copper, which was used as a 3D battery anode in a LIB [288].

### 6.4. Application in Thermal Insulations

Among different types of regenerated-cellulose-based materials, due to their highly porous nature that results in low thermal conductivity and their low density, aerogels are thought to have the potential to become a cost-effective and green solution for thermal insulation [290]. Regenerated cellulose aerogels exhibit thermal conductivity between 0.029 and 0.075 W/(m·K) [291], which is dependent on several preparation parameters, such as the solvent used for dissolution of cellulose and the amount of cellulose used [292,293]. Effective applications of cellulose-based aerogels for thermal insulators require their thermal conductivity, in principle, to be lower than 0.025 W/(m·K) at 300K and 1 atm—the thermal conductivity of “free” atmospheric air [294].

Lu et al. [295] showed that regenerated-cellulose-based aerogels (prepared from lignocellulosic biomass) can achieve lower thermal conductivities that could make them comparable to the traditional thermal insulators, such as glass wool, Styrofoam, and polyurethane foam. The authors reported that the lowest thermal conductivity of 0.030 W/(m·K) was achieved for an aerogel sample with the highest surface area among their samples. Nguyen et al. [292] prepared regenerated cellulose aerogel from a 2% cellulose solution and investigated its thermal insulation ability. The prepared aerogel exhibited thermal conductivity of 0.032 W/(m·K), which is comparable to some common commercial insulation materials such as mineral wool (0.03–0.04 W/(m·K)) [296]. In another study, Shi et al. [297] reported that the thermal conductivity of cellulose aerogels varied from 0.032 to 0.046 W/(m·K) depending on the dissolution and regeneration conditions for the preparation of aerogels. An aerogel with a somewhat lower thermal conductivity (0.032 W/(m·K)) could have application potential as an insulation material in fields where low or medium thermal insulation is required. Similarly, Karadalgi et al. [293] prepared regenerated cellulose aerogel fibers from solutions of different cellulose concentrations. The measurement of the thermal conductivity of the samples showed that it linearly increased from 0.04 to 0.075 W/(m·K) as the cellulose content of the respective solution increased. As these studies showed, regenerated-cellulose-based aerogels show comparable or slightly lower thermal conductivity than traditional thermal insulation materials such as mineral/glass wool and expanded polystyrene (0.032–0.037 W/(m·K)) and wood or hemp fiber insulation boards (~0.04 W/(m·K)). However, for their potential commercial application, they need to have substantially lower thermal conductivities than the far less expensive conventional solutions to offset cost-related issues [298]. Furthermore, performance-related concerns such as moisture sensitivity, flammability, and thermal stability need to be addressed [290].

Studies have shown that the silane-coating or covalent-salinization of cellulose-based aerogels using methyltrichlorosilane (MTS) is effective for hydrophobic surface modification [299,300]. Interestingly, Nguyen et al. [292] showed that the MTS coating of a regenerated cellulose aerogel not only imparted hydrophobic characteristics but also led to a decrease in thermal conductivity; the thermal conductivity of the aerogel was reduced from 0.032 to 0.029 W/(m·K) once it was coated with MTS.

The incorporation of metal and metalloid hydroxide nanoparticles such as magnesium and aluminum hydroxide in the cellulose gel matrix during the sol–gel process has been investigated as a means to fabricate flame-retardant regenerated cellulose aerogels [301,302]. Han et al. [301] fabricated regenerated cellulose aerogel with excellent flame-retardancy via the non-agglomerated growth of magnesium hydroxide in situ in a cellulose gel matrix followed by freeze-drying. However, there was a penalty of increased thermal conductivity, which “moderately” increased from 0.056 to 0.081 W/(m·K).

Silica aerogel products, due to their ultralow thermal conductivity of ~0.015 W/(m·K), have already entered niche markets in building and pipe insulation industries as super thermal insulators, albeit with a poor mechanical strength that is considered to be one of the major constraints for industrial scale applications [298,303]. In their effort to prepare mechanically strong aerogels with high thermal insulating ability, researchers have studied regenerated-cellulose–silica composites aerogels [296,304,305,306]. Demilecamps et al. [304] prepared composite aerogels with interpenetrated cellulose–silica networks. The composite aerogels displayed a lower thermal conductivity (0.026 W/(m·K)) compared to the control cellulose aerogel (0.033 W/(m·K)). The mechanical strength (Young’s modulus) of the composite aerogel was improved in comparison to control aerogels (both cellulose and silica). However, compared to pure silica aerogel, there was a large penalty of thermal conductivity from 15 to 25 W/(m·K).

As summarized above, regenerated cellulose (pure or composite) has low thermal conductivity and relatively strong mechanical strength, thus indicating great potential in thermal insulation applications. Nonetheless, the development of regenerated-cellulose-based thermal insulation applications is constrained by cellulose-based aerogels’ higher thermal conductivity compared to conventional aerogels such as silica, as well as the inherently high cost of preparation.

In addition to all aforementioned applications, cellulose-inspired bioproducts have shown great potential in agriculture [307]. Most of these are cellulose derivatives and not pure cellulosic materials, so they are not discussed in this review in detail. However, we would like to briefly mention the agricultural systems for use in pest management [308,309], which are replacing materials such as plastic mulches, synthetic fertilizers, and pesticides/herbicides that have negatively impacted environmental and human health [307]. These materials have been perceived as important products suitable to minimize the impact of harmful practices and ensure the sustainability of agriculture.

## 7. Conclusions

Cellulose is a vitally important natural biopolymer that holds great potential as a raw material to realize the long-sought goal of preparing economically viable and equally performing “green” substitutes to synthetic polymer-based commodity products. The efficient and environmentally friendly dissolution of cellulose, which remains a nontrivial task, is crucial for its transformation into the desired types of polymeric commodity products. Due to its importance, cellulose dissolution is an active area of research. In this review, we have presented recent developments on cellulose dissolution while focusing on some widely used cellulose solvents, specifically NaOH-based, DMAC/LiCl, NMMO, and ILs. Different forms of regenerated cellulose products such as films, fibers, hydrogels, and aerogels, as well as their application in biomedicine, sorption, energy, and thermal insulation, have been reviewed.

## Figures and Tables

**Table 1:** Representative examples of cellulose dissolution in NaOH-based solvents.

Solvent System	Cellulose Source	Pretreatment	Effects on Cellulose	Dissolution Conditions			Ref.
				Conc. (wt%)	Temp. (°C)	Time (h)	
NaOH/H_2_O							
NaOH/H_2_O (9.1:90.9 *w*/*w*)	Soft wood and hard wood pulps, DP 1060; 994	Steam explosion	Decrease DP DP 180; 287	5	4	8	[82]
NaOH/H_2_O (8:92 *w*/*w*)	Dissolving sulfite wood pulps; DP 2375; 1410	Enzymatic (Cellulase + Econase HC400)	Digestion of primary cell wall, Decrease DP DP 925; 590	0.7 ^a^	−6	2	[85]
NaOH/H_2_O (7.6:92.4 *w*/*w*)	Commercial MCC; DP ~170	None	NA	7.6	−6	Footnote ^b^	[66]
NaOH/Urea/H_2_O							
NaOH/urea/H_2_O (7:12:81 *w*/*w*)	Commercial softwood unbleached Kraft pulp; DP 1300	Ball milling	Decrease DP and crystallinity DP 330, CrI ~0%	1	−12	>24	[86]
NaOH/Urea/H_2_O (7:12:81 *w*/*w*)	Commercial dissolving pulp; DP ~750	Ethanol–hydrochloric acid	Decrease DP, weakening cell wall DP ~190	4	−10	>2	[47]
NaOH/Urea/H_2_O (6:4:90 *w*/*w*)	Cotton linters; DP 850	Enzymatic (Celluclast)	Decrease DP DP 620–680	1.2 ^c^	−15	4	[84]
NaOH/Urea/H_2_O (6:4:90 *w*/*w*)	Cotton linters; DP ~410	None	NA	5	0	12	[77]
NaOH/Urea/H_2_O (7:12:81 *w*/*w*)	Cotton linters; DP 570	None	NA	4	0	Footnote ^d^	[87]
NaOH/Urea/H_2_O (7:12:81 *w*/*w*)	Cotton linters; DP ~620	None	NA	4	−10	0.1	[88]
NaOH/Urea/ZnO/H_2_O							
NaOH/Urea/ ZnO/H_2_O (10.2:4.2:0.8:84.8 *w*/*w*)	Commercial cellulose pulp, DP 553	Hydrothermal	Decrease DP, decrease polydispersity (PDI) DP 290–405	7.45	2	0.2	[81]
NaOH/Urea/ ZnO/H_2_O (7:12:0.5:80.5 *w*/*w*)	Commercial cotton linter pulp; DP ~1050	None	NA	2.5	−13	2	[89]
NaOH/Urea/Thiourea/H_2_O							
NaOH/Urea/ Thiourea/H_2_O (8:8:6:78 *w*/*w*)	Dissolving pulps; DP 780	Steam explosion	Decrease DP DP ~150	5	−10	60 Footnote ^e^	[90]
NaOH/Thiourea/ H_2_O (9.5:4.5:86 *w*/*w*)	Pulp sheets of cotton linters; DP 330; 620	None	NA	5, 7.5	−8; −10	0.1	[91]
Other Systems							
NaOH/PEG/H_2_O (9:1:90 *w*/*w*)	Commercial cellulose powder; DP ~810	None	NA	13	−15	15	[92]
NaOH/TMAH/H_2_O (9.2:21.0:69.8 *w*/*w*) ^f^	Commercial MCC; DP ~180	None	NA	5	−20	Footnote ^g^	[93]

^a^ 1 wt.% of cellulose was placed into solution, but solubility equaled 70% of loaded biopolymer. ^b^ Cellulose was swelled in water at 5 °C for 2 h followed by mixing in a pre-cooled NaOH solution at −6, and the mixture was stirred at −6 °C for 2 h. ^c^ 2 wt.% of cellulose were placed into solution, but solubility equaled 60% of loaded biopolymer. NA—no changes detected.^d^ Cellulose was dispersed in a 14 wt% NaOH solution at 0 °C for 1 min, then an equal amount (*w*/*w*) of pre-cooled to 0 °C 28% urea solution was added, and then the mixture was vigorously stirred for 2 min. ^e^ The pulp was added to the solvent system pre-cooled at −10 °C, followed by the vigorous stirring of the mixture for 20 min in an ice bath then storage at 3 °C for 60 h. ^f^ TMAH = Tetramethylammonium hydroxide. Weight percent recalculated from 2.3 M 50/50 mol/mol NaOH/TMAH. ^g^ Cellulose was placed into the solvent and stirred for 5 min in an ice bath, followed by freezing at −20 °C for 20 min, and then stirring in an ice bath for 5–30 min.

**Table 3:** Representative examples of cellulose dissolution in ILs.

Solvent Systems	Cellulose (Mw) or Degree of Polymerization (DP)	Load, wt.%	Dissolution Conditions	Ref.
[C_4_mim]Cl	Cellulose pulp (DP ≈ 1000)	10	Thermal dissolution at 100 °C	[150]
[C_4_mim]Cl	Cellulose pulp (DP ≈ 1000)	3	Thermal dissolution at 70 °C	[150]
[C_4_mim]Cl	Cellulose pulp (DP ≈ 1000)	5	Thermal dissolution at 80 °C + sonication	[150]
[C_4_mim]Cl	Cellulose pulp (DP ≈ 1000)	25	Microwave-assisted dissolution (at 100–130 °C) for a few minutes in 3–5 s pulses	[150]
[C_4_mim]Br	Cellulose pulp (DP ≈ 1000)	5–7	Microwave-assisted dissolution (at 100–130 °C) for a few minutes in 3–5 s pulses	[150]
[C_4_mim][SCN]	Cellulose pulp (DP ≈ 1000)	5–7	Microwave-assisted dissolution (at 100–130 °C) for a few minutes in 3–5 s pulses	[150]
[C_6_mim]Cl	Cellulose pulp (DP ≈ 1000)	5	Thermal dissolution at 100 °C	[150]
[C_2_C_1_im][(OMe)(H)PO_2_] with 1-alkylimidazoles (Alkyl = CH_3_, C_2_H_5_, C_3_H_7_, C_4_H_9_)	Scoured, bleached, air-dried, ground cotton fibers	5	Microwave-assisted dissolution followed by 90 °C oven: dissolved immediately after microwave or stored in oven up to 1 h	[162]
[C_4_C_1_im][(OMe)(H)PO_2_] with 1-alkylimidazoles (Alkyl = CH_3_, C_2_H_5_, C_3_H_7_, C_4_H_9_)	Scoured, bleached, air-dried, ground cotton fibers	5	Microwave-assisted dissolution followed by 90 °C oven: dissolved immediately after microwave or stored in oven up to 1 h	[162]
[(Bnz)_2_im][OAc] (Bnz = benzyl)	Scoured, bleached, air-dried, ground cotton fibers	5	Microwave-assisted dissolution followed by 90 °C oven: dissolved immediately after microwave or stored in oven up to 1 h	[40]
[NapmC_1_im][OAc] (Nap = Naphtalyl)	Scoured, bleached, air-dried, ground cotton fibers	5	Microwave-assisted dissolution followed by 90 °C oven: dissolved immediately after microwave or stored in oven up to 1 h	[40]
[C_4_mim]Cl	Avicel (DP 286), spruce sulfite pulp (dp-593), cotton Linters (dp-1198)	1–39	Thermal dissolution at 80 °C, 12 h	[163]
[C_4_mim][CH_3_COO]/DMSO = 2.54:1 mol/mol	MCC (DP 229)	15	Not provided	[164]
[C_4_mim][PhCOO]/DMSO = 2.54:1 mol/mol	MCC (DP 229)	9	Not provided	[164]
[C_2_mim][OAc]	Cotton pulp (the α-cellulose content 94%, DP 510)	16	90 °C oil bath for 7 h	[165]
[C_4_mim][OAc]	Cotton pulp (the α-cellulose content 94%, DP 510)	15	Thermal dissolution at 70 °C for 7 h	[165]
[C_2_mim]Cl	Cotton pulp (the α-cellulose content 94%, DP 510)	14	Thermal dissolution at 70 °C for 7 h	[165]
[C_4_mim]Cl	Cotton pulp (the α-cellulose content 94%, DP 510)	13	Thermal dissolution at 70 °C for 7 h	[165]
[C_4_mim][HSO_4_]	Cotton pulp (the α-cellulose content 94%, DP 510)	11	Thermal dissolution at 70 °C for 7 h	[165]
[C_4_mim][FeCl_4_]	Cotton pulp (the α-cellulose content 94%, DP 510)	<5	Thermal dissolution at 70 °C for 7 h	[165]
[C_4_mim]Br	Cotton pulp (the α-cellulose content 94%, DP 510)	<3	Thermal dissolution at 70 °C for 7 h	[165]
[C_2_mim]Br	Cotton pulp (the α-cellulose content 94%, DP 510)	<5	Thermal dissolution at 70 °C for 7 h	[165]
[C_2_mim]Cl/[C_2_mim][OAc] = 30:70 mol/mol	MCC	40	Stepwise thermal dissolution of cellulose at 100 °C to determine solubility	[166]
[C_2_mim]Cl/[C_4_mim]Cl eutectic mixture	MCC	35	Stepwise thermal dissolution of cellulose at 100 °C to determine solubility	[166]
[C_2_mim]Cl	MCC	12	Stepwise thermal dissolution of cellulose at 100 °C to determine solubility	[166]
[C_4_mim]Cl	MCC	29	Stepwise thermal dissolution of cellulose at 100 °C to determine solubility	[166]
[C_2_mim][OAc]	MCC	23	Stepwise thermal dissolution of cellulose at 100 °C to determine solubility	[166]
[C_4_mim]Cl	MCC	4.75	Microwave-assisted dissolution for a few minutes in 3–5 s pulses	[167]
[Amim]Cl	MCC	15	Thermal dissolution at 100 °C for 24 h	[187]
[C_4_mim]Cl	Bleached and dried softwood Kraft pulp sheets from a pulp mill	3–7.4	Thermal dissolution at 130 °C for 4 h	[188]
[C_4_mim]Cl	Cellulose powder (MW: 194,400)	1.5–4	Thermal dissolution at 80 °C for up to 40 min	[189]
[C_4_mim]Cl/DMSO	Cellulose powder (MW: 194,400)	4–5	Thermal dissolution at 80 °C for up to 40 min	[189]
[Amim]Cl	Wood pulp (α-cellulose 94.9%)	4	Thermal dissolution at 100 °C for ~45 min	[190]
[C6mim]Cl	Nanocrystalline cellulose	3	Thermal dissolution at 85 °C for 2 h	[191]
[C2mim][OAc]	Avicel PH-101 microcrystalline cellulose (MW = 160,000–560,000 g/mol)	2; 5	Thermal dissolution at 60 °C for 1 h	[192]
[mTBDH][OAc]	Cellulose of birch prehydrolysis kraft pulp (Enocell)	13	Thermal dissolution at a temperature of 85 and 80 °C and 15 mbar with stirring (30 rpm) for 75 min	[161]
[DBNH][OAc]	Cellulose of birch prehydrolysis kraft pulp (Enocell)	13	Thermal dissolution at a temperature of 85 and 80 °C and 15 mbar with stirring (30 rpm) for 75 min	[161]

## Data Availability

Not applicable.
